# The psychological impact of conflict in the Middle East from 2023 to 2025 on Australian communities: a prospective cohort study

**DOI:** 10.1016/j.lanwpc.2025.101639

**Published:** 2025-07-22

**Authors:** Susan J. Rees, Tyson Whitten, Alvin Kuowei Tay, Aino Suomi, Batool Moussa, Fatima Hassoun, Nawal Nadar, Derrick Silove

**Affiliations:** a Discipline of Psychiatry and Mental Health, School of Clinical Medicine, Faculty of Medicine and Health, University of New South Wales, Australia; b Childlight (East Asia Pacific), School of Social Sciences, University of New South Wales, Australia; c POLIS: The Centre for Social Policy Research, Australian National University, Australia

**Keywords:** Middle East conflict, Mental health, Panic disorder, High-income country, Quality of life, Longitudinal

## Abstract

**Background:**

There is a lack of empirical research on the mental health risks faced by populations living in high-income multicultural countries during a war in their country of origin. We examined mental health and psychosocial outcomes associated with a period during the 2023–2025 Middle East conflict (primarily involving Israel, Palestine and Lebanon) on Australian resident women including those who arrived from Lebanon, Gaza and other Palestinian Territories.

**Methods:**

The mental health study assessed 410 Australian resident women at two points: one 12–18 months prior, and one period during the current Middle Eastern conflict extending from October 7, 2023, to December 2024. The three groups included those directly connected by birth or family to the conflict-affected regions: Lebanon, Gaza and other Palestinian territories (Middle East—LGP), Other Migrants not from the region, and Australian Born (AB) women with no connection to the region. Measures included the Mini-International Neuropsychiatric Interview to assess symptoms of panic disorder (PD), mood disorder (MDD), post-traumatic stress disorder (PTSD), separation anxiety disorder (SEPAD), Quality of Life, Worry about Family and Separation from Family overseas. Generalised linear mixed models and cumulative link mixed models were used to examine the trajectory of mental disorder symptoms over time for each group compared with the AB group. The analysis adjusted for age, marital status, financial difficulties, and COVID-19 stress.

**Findings:**

Generalised linear and cumulative link mixed models revealed significant interaction effects, indicating that Middle East–LGP women experienced a significantly greater increase in PD symptoms (β = 1.26, SE = 0.54, *p* = 0.02) and poorer quality of life (β = 0.10, SE = 0.04, *p* = 0.009) from Time 1 to Time 2 compared to AB women. The Middle East—LGP women reported significantly greater increases in concerns about family overseas (log odds = 4.04, SE = 1.25, *p* = 0.001) and the ability to return home in an emergency (log odds = 3.41, SE = 1.20, *p* = 0.005).

**Interpretation:**

This is a unique study of women’s mental health in a multicultural, high-income country, undertaken during conflict occurring in another region of the world. Panic Disorder symptoms, poorer quality of life and other psychosocial stress increased only in the group connected by migration to the conflict-affected region.

**Funding:**

10.13039/501100000925National Health and Medical Research Council, Australia (2018/GNT1164736).

**Research in context**

**Evidence before this study**

There is a published commentary and growing concerns about the mental health and wellbeing of people from the Middle East region living in Australia during the current conflict. There is a lack of longitudinal evidence on the mental health and psychosocial impacts of war or conflict in Middle Eastern and other diaspora populations with connection by birth or parental migration.

**Added value of this study**

This is the first longitudinal study to examine the mental health and psychosocial impact of conflict occurring in another country, with evidence drawing attention to panic disorder symptoms, worry about family and poorer quality of life for resident women with personal or family connections to the impacted region. The study demonstrates that timely and targeted mental health and social support is needed, and for international communities and decision-makers to de-escalate conflict and uphold human rights and international laws that aim to protect civilians from harm.

**Implications of all the available evidence**

The Middle East war is having a significant impact on the mental health of Australian women, and urgent attention needs to be given to those who have migrated or have family from the directly impacted countries. Practitioners should be specifically trained to screen and treat panic symptoms in women with personal or familial ties to Lebanon, Gaza, or other parts of the Palestinian Territories. Intervening to address acute responses during conflict in another country may reduce long-term mental health and quality of life sequelae amongst communities in high-income multicultural countries.

## Introduction

Armed conflicts erupted in Israel, the Gaza Strip and Lebanon in October 2023. The severity and horror of the conflict has been extensively documented by the Australian and international news and social media. This study focuses on women from Gaza, other Palestinian Territories, and Lebanon who reside in Australia because there are compelling reasons to anticipate adverse mental health and psychosocial consequences linked to exposure to news of mass deaths, casualties, and widespread human rights violations (including displacement, rape, starvation and destruction of homes and infrastructure) inflicted on these populations.^1^, ^2^, ^3^, ^4^, ^5^ Whilst evidence shows negative mental health impacts of war on conflict-affected populations and veterans, knowledge of the extent and type of mental health responses to extremely distressing conflicts amongst people living outside a conflict-affected region of origin or familial association is lacking.^6^, ^7^, ^8^, ^9^

Exposure to war and conflict before arrival in the settlement country is associated with mental disorders and problems with daily functioning in migrants and refugees.^10^, ^11^, ^12^ Research suggests that conflict-affected people have higher rates of mental disorders than the general population, with most studies examining depressive disorder and PTSD.^7^ Longitudinal studies and systematic reviews are mixed in relation to whether PTSD and depressive responses tend to improve, persist or worsen after resettlement, however there is consensus that pre-migration traumas, as well post-migration stressors, including separation from family and financial difficulties, are significant factors in shaping mental health status.^7^ A noted shortcoming is that no studies have examined the possible impact of recurrent conflict occurring in countries of personal or family origin on potentially vulnerable migrant populations.

High-income countries including Australia welcome large numbers of migrants and refugees each year. In 2021, Australia was home to approximately 200,000 people born in the Middle East. Over 248,000 Australians reported Lebanese ancestry and 800,000 identified as having family connections to Arabic-speaking countries in the Middle East.^13^ Factors including vicarious and intergenerational trauma, along with heightened feelings of empathy, may increase mental health risks amongst populations that were not only born in the conflict-affected country but also have family members currently living or born there.^9^^,^^14^ Migrants from other countries may also be at risk because of their exposure to war trauma prior to settlement.^10^^,^^11^ Failure to respond to acute mental health responses in those who identify closely with regions in conflict could have immediate as well as long-term mental health repercussions, including diagnosed mental disorders and impaired quality of life.^15^

Women are at greater risk of mental disorder, including during and following traumatic events, compared to men,^16^, ^17^, ^18^ a difference that extends to women from migrant and refugee backgrounds.^19^ In this study we aimed to document the mental health and psychosocial impacts of the Middle East conflict during 2023–2024 on Australian resident women, approximately one-fifth of whom identified as being connected directly or by birth of their parents to any of three conflict-affected locations (Lebanon, Gaza and other Palestinian Territories), henceforth Middle East—LGP group.

We hypothesised that the Middle East–LGP group would have an increase in symptoms of panic disorder (PD), adult separation anxiety disorder (SEPAD), Major Depressive Disorder (MDD), post-traumatic stress disorder (PTSD) as well as deterioration in quality of life, and stress related to worry about family overseas, separation from family, and being unable to return home in an emergency.^20^, ^21^, ^22^, ^23^ We further hypothesised that Other Migrants, including those who had previously fled war and conflict and those born in Australia to migrant parents other than Lebanon, Gaza and Palestine, would have increased mental disorder symptoms and psychosocial problems when compared to the Australian-born group with no parental history of migration, although not to the same extent as the Middle East-LGP group.^11^

## Methods

### Sample

Data were drawn from the Women Aware with Their Children (WATCH) study, an ongoing longitudinal mental health study of 1335 women (685 consecutively enrolled from refugee backgrounds and 650 randomly selected Australian-born including second generation migrants) recruited in 2015 and 2016 from three public antenatal clinics in Sydney and Melbourne, Australia.^22^^,^^23^ All participants were fully informed and provided consent prior to participating in the study. Consecutive enrolment is where all subjects meeting the inclusion criteria are approached for participation, and it can minimise bias when studying minority or ‘hidden’ populations. The Australian born (AB) population was larger in number, and therefore we elected to use a kish grid to randomise who we approached based on the number of women attending the clinic each day of the study.^23^ We recruited from all Arabic-speaking countries, Sudan (all regions of Sudan) and Sri Lanka (Tamil-speaking). These nations represented the largest intake groups from conflict-affected regions entering Australia and other high-income countries at the time of this study. By limiting the study to these language groups, we sought to contain both the problems of transcultural measurement error and small cell sizes. Country of origin was determined using clinic records, documented requests for an interpreter, culturally recognisable surnames, and by cross-referencing country of birth data with the clinic appointment list.

The WATCH study is unique in that all interviews at each timepoint are undertaken by a same language speaking research assistant. Six interview timepoints have been undertaken, at 12–18-month intervals, with 65.7% retention and low risk of attrition bias. Some individuals did not participate in follow-up interviews due to time constraints or because they could not be contacted despite repeated attempts.^22^ Data for the current study were based on interviews conducted at Time 5 (August 2021–March 2023), that is prior to the current Middle East conflict, and Time 6 (July 2023–December 2024) that is, during the period of the Middle East conflict that started on October 7, 2023, herein referred to as Time 1 and 2. Of the 544 women interviewed at both time points, 134 had their Time 2 interview prior to commencement of conflict on October 7; these women were excluded from the analyses. One participant was from Israel and was excluded from the present analysis due to insufficient sample size. Hence, the analytical sample for the current study comprised of 410 women. All age groups were included, noting they are women of childbearing age. All women had been in Australia for at least 8 years.

#### Groups

Country of birth and origin of parents were documented at the baseline interview. Those born in Australia with both parents originating from that country were designated to the reference group “*Australian-born*” (AB). Women were designated as “*Middle East—Lebanon, Gaza, Palestine”* (Middle East–LGP) if either they or at least one of their parents were born in the three directly involved areas of Lebanon, Gaza, or Palestine. Women who indicated that they or at least one of their parents were born outside of Australia, Lebanon, Gaza or Palestine were designated “Other Migrant.” The Other Migrant group is included because of the potential for vicarious stress reactions of migrants exposed to war and conflict in countries other than Lebanon, Gaza or Palestine.^14^ This is particularly likely if they had personal exposure to conflict prior to arrival in the settlement country. Our sample of Other Migrants, consistent with multicultural countries that welcome refugees and migrants, included approximately half (53%) who had fled conflict-affected countries, incorporating those from close proximity to the current conflict.^11^ Parental birth was also an inclusion criterion because residents with parents who arrived as migrants theoretically share emotional empathy and cultural identification with the parent’s compatriots and country, and may indirectly become psychologically impacted through mechanisms of vicarious and intergenerational trauma and other affective responses.^24^

### Measures

#### Mental health symptoms

Outcomes commonly associated with war trauma were measured at both Time 1 and 2. The Mini-International Neuropsychiatric Interview (MINI) based on the Diagnostic and Statistical Manual of Mental Disorders (Fourth Edition) (DSM-IV) was used for each disorder. A public health approach was employed, where each measure was scored and aggregated to produce a continuous score. Participants received a score of 0 if no symptoms were endorsed. Each endorsed symptom was assigned a value of 1, resulting in a cumulative symptom count per participant. Descriptive statistics, including means and standard deviations, were calculated for each disorder to summarise symptom burden across the sample. We assessed the number of symptoms relating to Major Depressive Disorder (MDD; 9 items, score range 0–9; Time 1/2 a = 0.70/0.74), Panic Disorder (PD; 17 items, score range 0–17; Time 1/2 a = 0.83/0.85), Separation Anxiety Disorder (SEPAD); 14 items, score range 0–14, diagnostic threshold score = 3; Time 1/2 a = 0.84/0.81), and Post-Traumatic Stress Disorder (PTSD; 16 items, score range 0–16; Time 1/2 a = 0.92/0.90).^25^

Quality of life (QoL) comprised 15 items measuring self-reported physical, mental, and social difficulties, with responses recorded on a three-point Likert scale.^26^ Scores were averaged across all items, with higher values indicating poorer quality of life (Time 1/2 a = 0.79/0.82).

The Living Difficulties Checklist has been validated across multiple studies and was adapted for the WATCH study.^22^^,^^27^ Only participants from conflict-affected backgrounds, other migrants, and Australian-born who had lived overseas completed this measure (because it was specifically designed for those with migratory life experiences). Respondents were asked whether, in the past 12 months, they had experienced (i) worry about family overseas, (ii) separation from family, and (iii) being unable to return home in an emergency. They scored for each item (0) no problem at all, (1) a problem, or a (2) very serious problem.

#### Covariates

Maternal age in years and marital status (0 = never married, widowed, divorced, or separated; 1 = married or de facto relationship) were measured at Time 2. It was important to control for likely confounding factors. We included the number of financial stressors in the last 12 months (range 0–7) measured at Time 1 (a = 0.83) and Time 2 (a = 0.78). COVID difficulties collected at Time 1 and Time 2 were measured by averaging the scores across three items asking participants if COVID related (i) *hardship*, (ii) *fear or stress*, and (iii) *family conflict* was (0) no problem at all, (1) a problem, or a (2) very serious problem (Time 1/2 a = 0.41/0.71).^22^^,^^28^^,^^29^

### Ethics approval

The study is approved by the Southwestern Sydney Local Health District Human Research and Monash Health Ethics Committees (2019/ETH04272).

### Role of the funding source

The funders (NHMRC, Australia, 2018/GNT1164736) had no role in study design, data collection, data analysis, interpretation or writing of the manuscript.

### Analysis

Descriptive statistics were calculated for all study variables separately for women in the AB, Other Migrant and Middle East—LGP groups. To test within-group differences from Time 1 and 2 we used the non-parametric Wilcoxon signed-rank test. Between group differences were assessed using Welch’s ANOVA and chi-square test. A series of multivariate mixed model analyses were conducted to estimate the difference for each separate mental health outcome from Time 1 to 2 for the Other Migrant and Middle East–LGP groups, relative to AB women. For all women (n = 410), generalised linear mixed models (GLMM) were calculated for SEPAD (model 1), MDD (model 2), PD (model 3), PTSD (model 4), and QoL (model 5). Cumulative link mixed models (CLMM) (also known as ordinal Logistic Regression Models) were calculated for Middle East—LPG, Other Migrant and the Australian-born women who had lived overseas (n = 194) regarding their worries about family overseas (model 6), difficulty being separated from family (model 7), and concern about being unable to return home in an emergency (model 8). GLMMs analyse non-normally distributed outcomes with both fixed and random effects, accommodating clustered or repeated measures data. CLMMs extend this framework to ordinal outcomes by modelling cumulative probabilities across ordered categories.

Post-hoc simulation-based power analysis was conducted to estimate the minimum detectable significant (*p* < 0.05) interaction effect size in a repeated measures GLMM and CLMM, with simulation parameters mirroring that of the analytical sample. Based on 1000 Monte Carlo simulations, the GLMM (n = 410) could detect an interaction effect of β = 0.35 with 80.1% (95% CI: 77.5%–82.5%) statistical power, whereas the CLMM could detect β = 0.73 with 79.3% (95% CI: 76.8%–81.8%) power. Maternal age, marital status, financial difficulties (Time 1 and 2), and COVID difficulties (Times 1 and 2) were treated as fixed effects. Random intercepts were included for each participant. Continuous outcome data followed a lognormal distribution (i.e., right skewed). The relevant assumptions underpinning the GLMM and CLMM were assessed using diagnostic tools, including the DHARMa package for residual simulation and dispersion checks, Q–Q plots for evaluating the normality of random effects, and the performance package to assess multicollinearity and overall model fit; all assumptions were satisfied. Coefficients, standard errors, and *p* values (2-tailed) are presented. Analyses were conducted in R version 4.4.2 using the lme4, ordinal, lmerTest, and ggplot2 packages.^30^^,^^31^

## Results

Of the 410 women included in this study, 48.8% were designated as Australian-born, 34.1% Other Migrant, and 17.1% as Middle East–LGP. Regarding the latter group, most were of Lebanese heritage (87.1%). The average age for the total sample was 37.89 (sd = 5.40) years. Most women were married or in a de facto relationship (90.2%) and reported an average of 1.61 (sd = 1.24) and 1.60 (sd = 1.19) financial difficulties at Times 1 and 2. Descriptive statistics for each group, as well as between group comparisons, are presented in Table 1.

**Table 1:** Descriptive statistics at time 1 and 2 (n = 410).

	Time 1				Time 2			
	Australia born (n = 200)	Other migrant (n = 140)	Middle East—LGP (n = 70)	Between group comparison	Australia born (n = 200)	Other migrant (n = 140)	Middle East—LGP (n = 70)	Between group comparison
	Mean (sd)/n (%)	Mean (sd)/n (%)	Mean (sd)/n (%)		Mean (sd)/n (%)	Mean (sd)/n (%)	Mean (sd)/n (%)	
Age in years	–	–	–	–	38.11 (5.41)	37.64 (5.20)	37.75 (5.82)	*p* = 0.708
Married or de facto	–	–	–	–	173 (86.5%)	136 (95.8%)	61 (89.7%)	*p* = 0.017
Financial difficulties	1.447 (1.066)	1.758 (1.349)	1.78 (1.403)	*p* = 0.047	1.575 (1.184)	1.648 (1.21)	1.603 (1.174)	*p* = 0.856
Covid difficulties	0.551 (0.498)	0.596 (0.519)	0.616 (0.45)	*p* = 0.575	0.448 (0.558)	0.595 (0.555)	0.451 (0.552)	*p* = 0.044
SEPAD score	1.523 (3.535)	2.667 (4.347)	1.576 (3.592)	*p* = 0.027	1.325 (3.175)	2.239 (4.033)	1.25 (2.919)	*p* = 0.035
MDD score	0.82 (2.059)	1.227 (2.482)	0.881 (2.478)	*p* = 0.285	1.00 (2.425)	1.22 (2.355)	1.162 (2.531)	*p* = 0.694
PD score	1.80 (4.097)	0.747 (2.158)	0.544 (1.774)	*p* = 0.002	2.385 (4.71)	1.436 (3.824)	1.971 (4.35)	*p* = 0.144
PTSD score	0.92 (2.291)	1.303 (2.929)	0.397 (2.008)	*p* = 0.046	0.835 (2.514)	1.36 (3.104)	0.412 (2.002)	*p* = 0.041
QoL score	1.300 (0.277)	1.341 (0.29)	1.264 (0.408)	*p* = 0.208	1.338 (0.339)	1.367 (0.368)	1.435 (0.515)	*p* = 0.193
Worry about family overseas	0.583 (0.654)^a^	0.626 (0.835)^b^	0.632 (0.786)^c^	*p* = 0.969	0.042 (0.204)^a^	0.527 (0.797)^b^	0.895 (0.798)^c^	*p <* 0.001
Separation from family	0.25 (0.442)^a^	0.534 (0.798)^b^	0.231 (0.536)^c^	*p* = 0.028	0.083 (0.282)^a^	0.336 (0.663)^b^	0.282 (0.605)^c^	*p* = 0.185
Unable to return home	0.417 (0.654)^a^	0.515 (0.79)^b^	0.18 (0.556)^c^	*p* = 0.045	0.125 (0.448)^a^	0.215 (0.584)^b^	0.385 (0.711)^c^	*p* = 0.186

*p* values obtained from Welch’s ANOVA and chi-square test.

SEPAD, Separation Anxiety Disorder; MDD, Major Depressive Disorder; PD, Panic Disorder; PTSD, Post-Traumatic Stress Disorder; QoL, Quality of Life.

a n = 24.

b n = 130.

c n = 40.

The univariate within-group analysis demonstrated that the Middle East–LGP group had a significant increase in PD symptoms (*z* = 3.54, *p* < 0.001), poorer Quality of Life (QoL) (z = 4.52, *p* < 0.001), and concerns about being unable to return home in an emergency (*z* = 2.23, *p* = 0.03). For the Other Migrant group, there was a significant increase in PD scores (*z* = 2.63, *p* = 0.009), concern about being unable to return home in an emergency (*z* = 3.93, *p* < 0.001), and difficulty being separated from family (*z* = 3.22, *p* = 0.001). From Time 1 to 2, AB women also had a significant increase in PD scores (*z* = 2.11, *p* = 0.04), worry about family overseas (z = 3.13, *p* = 0.002), concerns about being unable to return home in an emergency (z = 2.33, *p* = 0.02), and difficulty being separated from family (*z* = 2.00, *p* = 0.05).

**Fig. 1:**
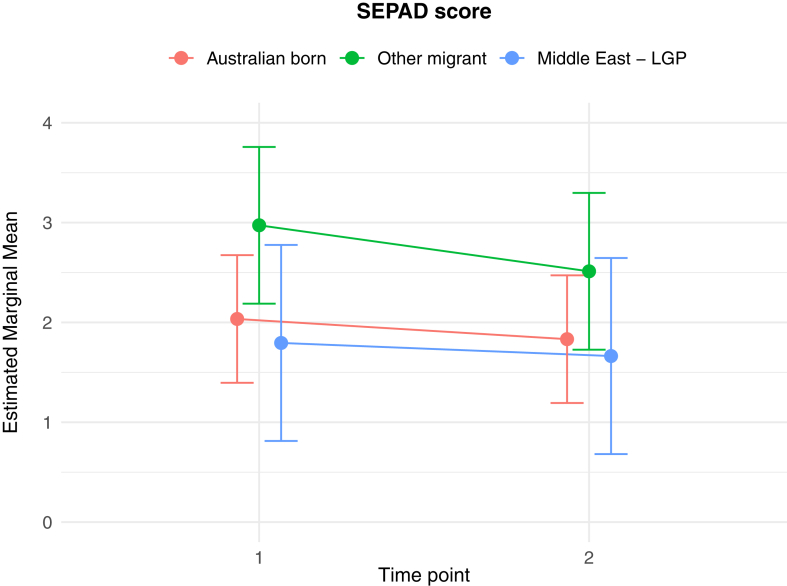
Estimated marginal means of SEPAD score.

**Fig. 2:**
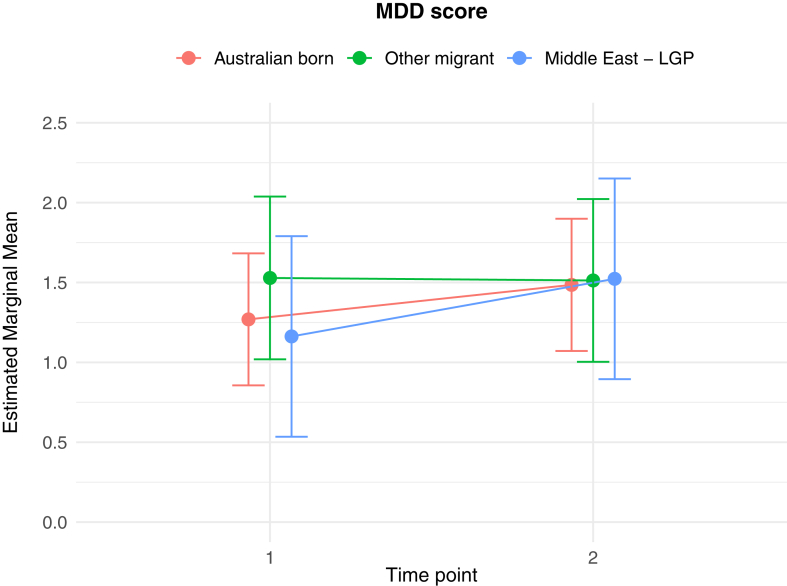
Estimated marginal means of MDD score.

**Fig. 3:**
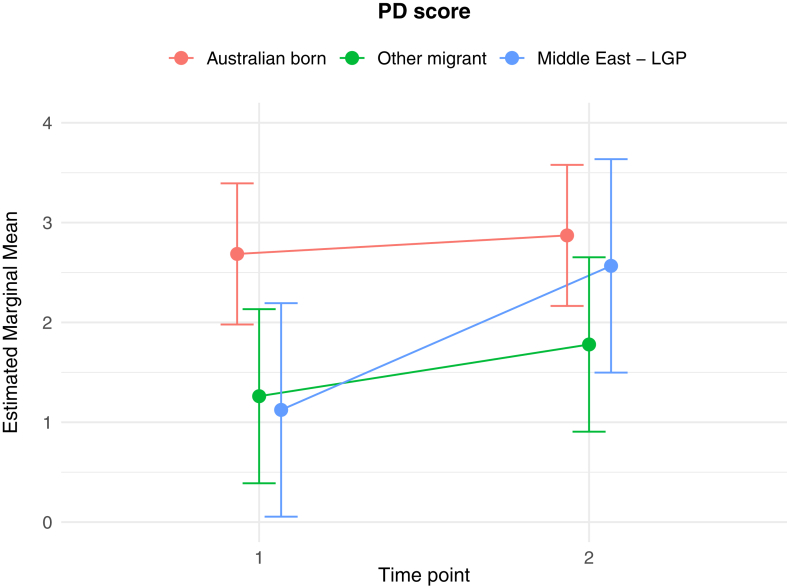
Estimated marginal means of PD score.

**Fig. 4:**
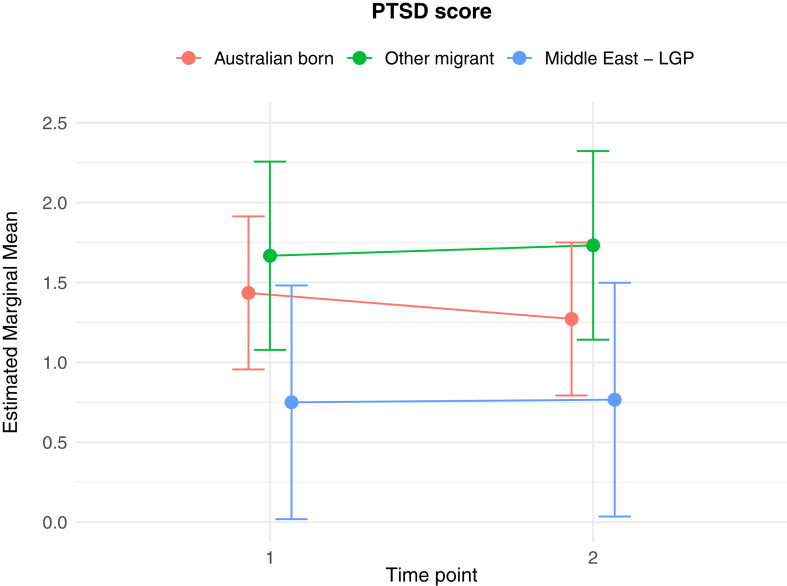
Estimated marginal means of PTSD score.

**Fig. 5:**
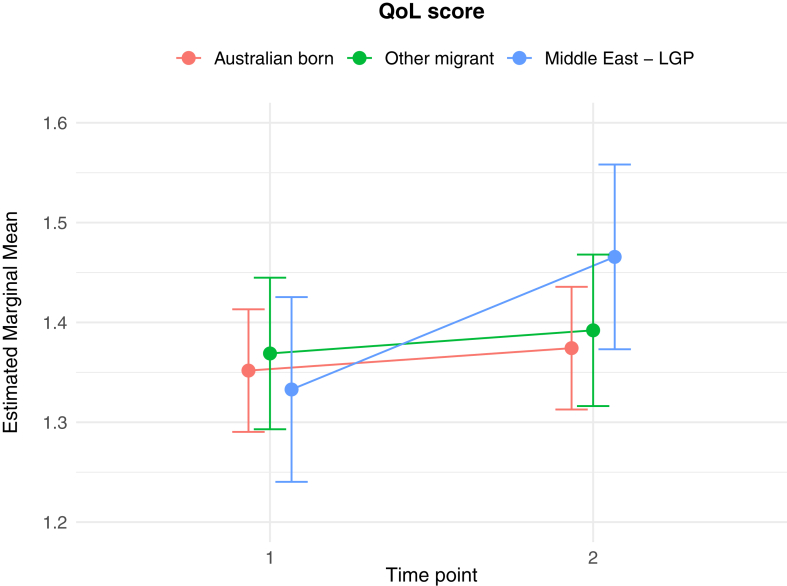
Estimated marginal means of QoL score.

**Fig. 6:**
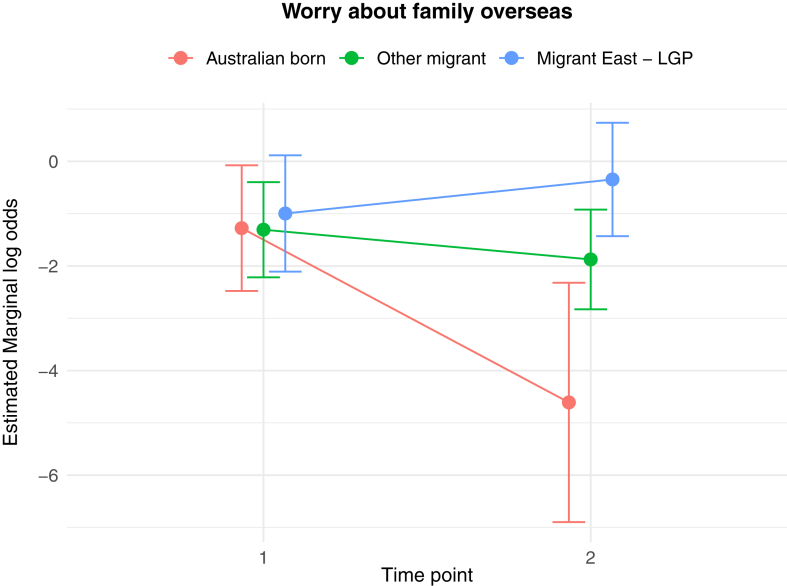
Estimated marginal log odds of worry about family overseas.

**Fig. 7:**
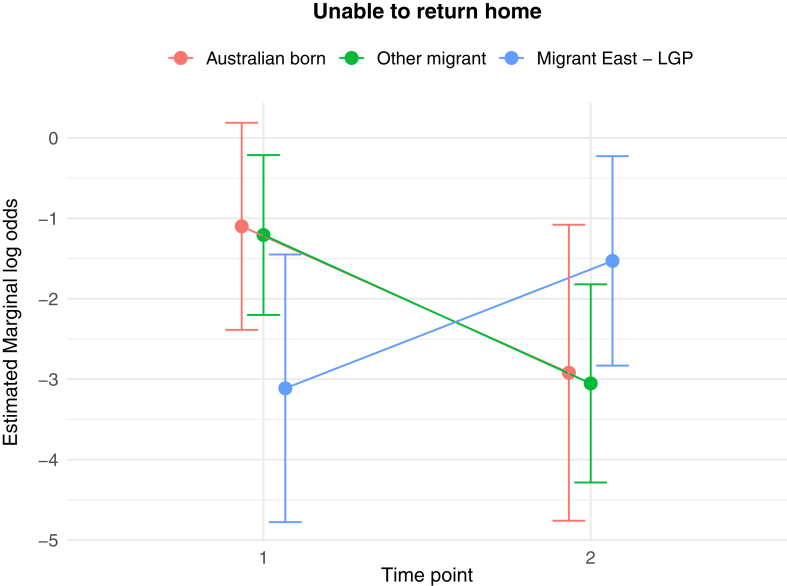
Estimated marginal log odds of unable to return home.

**Fig. 8:**
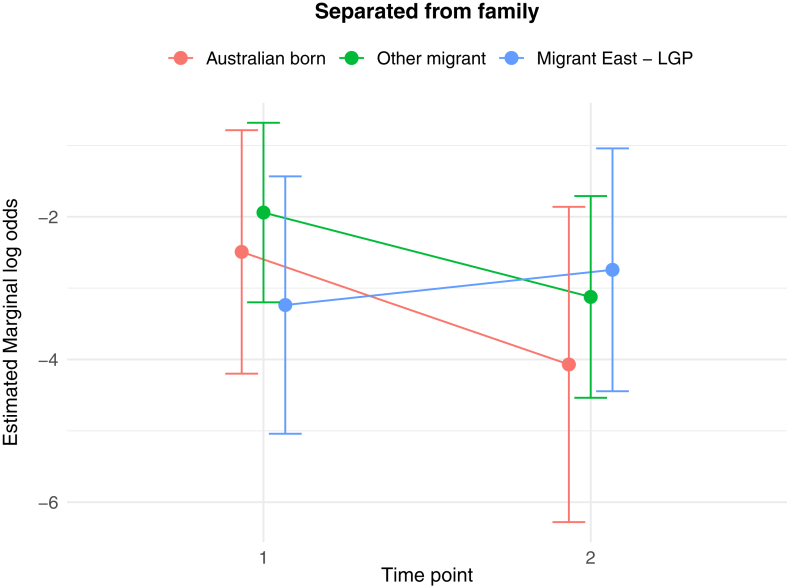
Estimated marginal log odds of separated from family.

The results of the generalised linear and cumulative link mixed models are presented in Table 2. Of key interest is the Middle East—LGP∗Time 2 interaction: coefficients in the GLMMs reflect the average change in outcome score, while the CLMMs indicate the average change in log odds of scoring in a higher or lower ordinal category, independent of other fixed and random effects. In relation to between group differences, interaction effects indicate significantly greater average increases from Time 1 to Time 2 in PD score (*b* = 1.26 (se = 0.54), *p* = 0.02), poorer QoL scores (*b* = 0.10 (se = 0.04), *p* = 0.009), worry about family overseas (log odds = 4.04 (se = 1.25), *p* = 0.001), and concerns about being unable to return home in an emergency (log odds = 3.41 (se = 1.20), *p* = 0.005), for the Middle East—LGP group, relative to AB. Fig. 1, Fig. 2, Fig. 3, Fig. 4, Fig. 5, Fig. 6, Fig. 7, Fig. 8 present the change in estimated marginal means (GLMM) and estimated marginal log odds (CLMM) for the respective models, and indicate that worry about family overseas decreased from Time 1 to 2 for both the AB and the Other Migrant group. However, this decrease was significantly greater for the AB than the Other Migrant group (log odds = 2.75 (se = 1.17), *p* = 0.02).

**Table 2:** Multivariate generalised linear and cumulative link mixed models (*β/*log odds (se)).

	Generalised linear mixed models (n = 410)					Cumulative link mixed models (n = 194)		
	SEPAD	MOOD	PD	PTSD	QoL	Worry about family overseas	Unable to return home	Separated from family
Intercept/threshold	−0.081 (1.212)	0.976 (0.818)	3.522 (1.416)	−0.656 (0.93)	1.009 (0.125)	0|1: 4.42 (1.55) 1|2: 5.87 (1.59)	0|1: 3.40 (1.69) 1|2: 4.54 (1.73)	0|1: 3.31 (2.07) 1|2: 5.20 (2.43)
Other migrant^a^	0.938 (0.406), *p* = 0.021	0.259 (0.256), *p* = 0.312	−1.425 (0.434), *p* < 0.01	0.233 (0.300), *p* = 0.438	0.017 (0.037), *p* = 0.646	−0.029 (0.554), *p* = 0.958	−0.108 (0.609), *p* = 0.859	0.60 (0.79), *p* = 0.447
Middle East—LGP^a^	−0.24 (0.514), *p* = 0.64	−0.107 (0.323), *p* = 0.741	−1.562 (0.547), *p* < 0.01	−0.685 (0.379), *p* = 0.072	0.019 (0.047), *p* = 0.688	0.173 (0.654), *p* = 0.791	−2.015 (0.874), *p* = 0.021	−0.757 (0.969), *p* = 0.435
Time 2	−0.202 (0.318), *p* = 0.525	0.216 (0.172), *p* = 0.210	0.19 (0.272), *p* = 0.496	−0.163 (0.218), *p* = 0.455	0.022 (0.021), *p* = 0.292	−3.303 (1.133), *p* = 0.004	−1.821 (0.921), *p* = 0.048	−1.596 (1.007), *p* = 0.113
Age	0.012 (0.028), *p* = 0.523	−0.019 (0.019), *p* = 0.321	−0.049 (0.032), *p* = 0.127	0.035 (0.021), *p* = 0.097	0.004 (0.003), *p* = 0.122	0.068 (0.032), *p* = 0.031	0.035 (0.036), *p* = 0.323	−0.02 (0.043), *p* = 0.648
Marital status	−0.958 (0.548), *p* = 0.081	−0.919 (0.371), *p* = 0.014	−1.357 (0.642), *p* = 0.035	−0.834 (0.421), *p* = 0.048	−0.010 (0.057), *p* = 0.087	0.502 (0.795), *p* = 0.528	−0.593 (0.869), *p* = 0.495	−0.08 (1.085), *p* = 0.941
Financial difficulties time 1	0.569 (0.149), *p* < 0.001	0.278 (0.101), *p* = 0.006	0.236 (0.175), *p* = 0.179	0.278 (0.115), *p* = 0.016	0.021 (0.015), *p* = 0.168	0.332 (0.151), *p* = 0.028	0.390 (0.168), *p* = 0.020	0.342 (0.21), *p* = 0.102
Financial difficulties time 2	0.117 (0.147), *p* = 0.425	0.305 (0.099), *p* = 0.002	0.334 (0.172), *p* = 0.053	0.160 (0.113), *p* = 0.159	0.065 (0.015), *p <* 0.001	−0.129 (0.189), *p* = 0.485	0.315 (0.197), *p* = 0.111	0.555 (0.25), *p* = 0.027
Covid difficulties time 1	1.127 (0.311), *p* < 0.001	0.743 (0.211), *p* < 0.001	0.37 (0.365), *p* = 0.311	0.337 (0.239), *p* = 0.159	0.101 (0.032), *p* < 0.003	0.717 (0.353), *p* = 0.042	0.840 (0.40), *p* = 0.036	1.110 (0.506), *p* = 0.029
Covid difficulties time 2	0.364 (0.292), *p* = 0.214	0.211 (0.198), *p* = 0.287	1.222 (0.342), *p* < 0.001	0.576 (0.224), *p* = 0.011	0.050 (0.031), *p* = 0.098	0.453 (0.332), *p* = 0.172	0.280 (0.375), *p* = 0.454	0.683 (0.459), *p* = 0.137
Other migrant^a^∗Time 2	−0.259 (0.491), *p* = 0.599	−0.233 (0.266), *p* = 0.384	0.333 (0.422), *p* = 0.42	0.228 (0.338), *p* = 0.500	0.001 (0.033), *p* = 0.983	2.745 (1.167), *p* = 0.019	−0.024 (0.992), *p* = 0.981	0.346 (1.065), *p* = 0.745
Middle East—LGP^a^∗Time 2	0.071 (0.629), *p* = 0.91	0.145 (0.340), *p* = 0.671	1.257 (0.539), *p* = 0.02	0.179 (0.431), *p* = 0.678	0.103 (0.042), *p* = 0.009	4.035 (1.25), *p* = 0.001	3.406 (1.203), *p* = 0.005	2.10 (1.23), *p* = 0.088
Random effects intercept	2.832 (1.683)	2.042 (1.429)	6.768 (2.602)	2.209 (1.486)	0.058 (0.241)	1.382 (1.175)	1.328 (1.153)	3.409 (1.846)

a Australian born women were the reference group.

## Discussion

Mental health and psychosocial wellbeing, including quality of life, in the Middle East—LGP and not the Other Migrant group appeared to be significantly impacted by the Middle East conflict, relative to the Australian-born group. We found an increase from Time 1 (before the conflict) to Time 2 (during the conflict) in panic disorder symptoms amongst the Middle East—LGP group. Despite panic disorder being one of the most common forms of mental illness in high-income countries, the bulk of the studies conducted with conflict-affected or forcibly displaced populations has focused primarily on categories of PTSD, depression, and anxiety in general.^8^^,^^32^^,^^33^ Where panic has been studied in conflict-affected populations, symptoms are often subsumed under PTSD, anxiety or anger categories.^34^^,^^35^ We report that panic disorder symptoms emerged on a trajectory quite independent from other disorders, and in direct response to conflict occurring in the Middle East.^36^^,^^37^ The findings for the Middle East–LGP group therefore suggest a pressing need for mental health identification and intervention for new onset panic disorder symptoms in this group residing in Australia—given the disabling effect of sudden onset acute anxiety involving both psychological and physiological symptoms. Other categories of mental health symptoms including major depressive disorder, separation anxiety and PTSD showed non-significant increases in the Middle East–LGP group. Insufficient statistical power may be partially responsible for this finding, so caution needs to be taken in assuming that an increase in these symptoms is not clinically important in the Middle East–LGP group.

Mental health practitioners need to be aware that panic symptoms during acute traumatic stress may be the harbinger for future mental ill-health including panic disorder, depression and PTSD.^37^^,^^38^ Because our findings relate specifically to the acute and ongoing phase of conflict, we recommend a stepwise trauma-informed approach using supportive psychotherapy, culturally appropriate community interventions, and practical support as the first-line intervention. Practitioners may then be required to undertake future clinical assessment for those who may have persisting, severe and disabling reactions.

The Middle East has experienced repeated cycles of instability, with multiple episodes of war and conflict preceding the current conflict examined in this study. Nevertheless, the current conflict is seen as one of the most devastating due to very high rates of death, injury, suffering and destruction, particularly impacting women and children, with calls for investigation into crimes against humanity, war crimes, and breaches of international humanitarian law.^2^^,^^5^^,^^39^ The finding that increased mental health symptomology and poorer quality of life (a measure of physical health, mental health and social-wellbeing domains) were associated with the onset of conflict in the relevant regions for the Middle East–LGP group was underscored by evidence of significantly greater ‘worry about family overseas’ and ‘being unable to return home in an emergency’, from Time 1 to Time 2. The pattern of change was substantially different compared with the Other Migrant and AB groups who had ever lived overseas, where these items decreased compared with the increase for the Middle East—LGP group.

The observable pattern of increase on all the three indices (i) worry about Family overseas, (ii) separation from family, and (iii) being unable to return home in an emergency for the Middle East LGP group is most likely because of vicarious exposure to conflict in the region they identify with. Although it increased, lack of statistical significance for ‘separation from family’ in the Middle East—LGP can be explained by limited statistical power. It is likely that, given the timing, the easing of COVID travel restrictions, access to COVID vaccines and improved health responses to COVID in many countries had an impact on the observable decrease in the three indices for the migrant and AB groups.

Our study signals a need for community-wide understanding and support for Australians with personal and family connections to ongoing conflict-impacted regions, and greater awareness of the mental distress experienced by separation and worry about family left behind in these situations.^40^ These rare longitudinal findings together reflect a plausible ‘trauma story’ for the Middle East–LGP group, which includes exposure to news of widespread death and harm and substantial worry about family overseas, most likely triggering the woman’s personal stress response in relation to her (or her family’s) past war-related trauma in those regions.^41^ In addition, we have noted concerns about an escalation in racial and religious prejudice against Arab populations in Australia, indicating the need for further research incorporating these stressors. Since data collection was completed, the conflict has persisted and intensified with increased attacks, loss of infrastructure and civilian deaths in Lebanon and an escalation in civilian deaths, mass starvation and injuries in Gaza. This suggests a high probability of increased mental health symptoms and psychosocial challenges amongst the Middle East–LGP group.^3^^,^^5^^,^^39^^,^^42^

Limitations include that, although this is a systematic cohort study, we do not claim representativeness of all Australians. Further, we cannot examine impacts for other genders or younger age people. As noted, there was only one Israel-born participant in the overall sample, precluding a focus on a population at high risk of adverse mental health consequences related to war trauma.^3^ Attacks on Israel and fear for the hostages, in combination with anti-Semitic attacks in Australia, suggests the need for mental health assessment of this population.^6^ We intentionally included a range of theoretically relevant psychosocial outcomes as per our hypothesis, and limited our co-variates to the key theoretically supported issues that would be confounding factors including COVID-19, financial stressors, and maternal age. A key strength of the WATCH study is its design, which from the outset in 2015 aimed to examine a range of theoretically relevant social and trauma factors impacting mental health over time. This included both standard diagnostic mental health measures and specific items on worry about family overseas, separation from family, and being unable to return home in an emergency.

Our two experimental groups included both Australians who arrived as migrants or refugees and Australians born to parents who had migrated from another country. We did not examine whether the mental health outcomes were worse for those born outside Australia, or for those whose parents migrated to Australia. We did not extend the analysis to examining comorbidity of disorders to avoid excessive complexity in reporting the findings, although we acknowledge that symptom overlap is commonly identified amongst the disorders assessed. We note that time since arrival can impact levels of trauma in recently arrived migrants and refugees, but our groups had all been resident in Australia for at least eight years. We also note that the AB group had a high rate of panic disorder symptoms, reflecting the known prevalence of this and most other anxiety disorders in high income countries, including for women in this age group.^43^

In conclusion, our study offers a wider perspective on the mental health of migrant populations by including members who arrived from, and also those with family ties in, regions of renewed conflict, specifically, including migrants and refugees born in conflict-affected countries and their offspring born in countries of resettlement. It is the first longitudinal study to allow a naturalistic examination of the mental health impact of conflict occurring in the country of origin on Australian communities. The study indicates the need for multicultural societies to be informed and prepared to identify and support at-risk groups during periods of war or conflict in their countries of origin. Urgent attention needs to be given to those who have migrated or have family from the directly impacted countries of Lebanon, Gaza and other Palestinian Territories. The 2023–2025 Middle East conflict is having a significant impact on their mental health, requiring a special focus on the provision of psychosocial support and where necessary clinical intervention. Intervening in settlement countries to address acute psychological reactions, particularly panic symptoms, during periods of war in another country may reduce long-term mental health sequelae and associated negative impact on day-to-day functioning.

Our findings underscore the importance of mental health professionals recognising and addressing the broader structural and humanitarian factors that contribute to psychological distress. This includes supporting efforts to uphold international human rights and humanitarian law, advocating to protect civilians, and acting to prevent war and conflict-related violence and suffering.^3^^,^^5^^,^^39^

## Contributors

All authors had full access to all the data in the study and take responsibility for the decision to submit it for publication. Susan Rees, Tyson Whitten, Alvin Kuowei Tay and Aino Suomi directly accessed and verified the data. Conceptualisation: Susan Rees, Derrick Silove, Fatima Hassoun, Batool Moussa, Nawal Nadar. Data curation: Susan Rees, Fatima Hassoun, Batool Moussa, Nawal Nadar. Formal analysis: Tyson Whitten, Alvin Kuowei Tay, Aino Suomi, Susan Rees. Funding acquisition: Susan Rees Investigation methodology: Susan Rees, Derrick Silove, Fatima Hassoun, Batool Moussa, Nawal Nadar. Project administration: Fatima Hassoun, Batool Moussa, Susan Rees. Resources and software: Susan Rees. Supervision: Susan Rees. Validation: Susan Rees, Tyson Whitten, Alvin Kuowei Tay, Aino Suomi. Visualisation: All authors contributed equally. Writing the original draft: All authors contributed equally. Draft review & editing: Susan Rees, Derrick Silove, Tyson Whitten, Alvin Kuowei Tay, Aino Suomi, Batool Moussa.

## Data sharing statement

Data are accessible on request made to corresponding authors or to the Australian Data Archive (https://ada.edu.au/).

## Declaration of interests

We declare no competing interests.
